# Vitamin D_3_ affects liver expression of pro-/anti-inflammatory cytokines and nitric oxide synthases in type 2 diabetes

**DOI:** 10.3389/ebm.2025.10456

**Published:** 2025-07-24

**Authors:** Ihor Shymanskyi, Olha Lisakovska, Mykola Veliky, Olha Mezhenska, Vasyl Bilous, Andrii Siromolot, Anna Khomenko, Dmytro Labudzynskyi, Tetyana Horid’ko, Elvira Pasichna

**Affiliations:** ^1^ Department of Biochemistry of Vitamins and Coenzymes, Palladin Institute of Biochemistry of the National Academy of Sciences of Ukraine, Kyiv, Ukraine; ^2^ Department of Enzyme Chemistry and Biochemistry, Palladin Institute of Biochemistry of the National Academy of Sciences of Ukraine, Kyiv, Ukraine; ^3^ Department of Molecular Immunology, Palladin Institute of Biochemistry of the National Academy of Sciences of Ukraine, Kyiv, Ukraine; ^4^ Department of Lipids Biochemistry, Palladin Institute of Biochemistry of the National Academy of Sciences of Ukraine, Kyiv, Ukraine

**Keywords:** type 2 diabetes mellitus, liver dysfunction, vitamin D_3_, nitric oxide, oxidative-nitrosative stress, inflammatory cytokines

## Abstract

Our objective was to study the effect of vitamin D_3_ (VD) on hepatocellular oxidative-nitrosative stress and pro/anti-inflammatory cytokines in relation to nitric oxide (NO) formation and NO synthase (NOS) levels in type 2 diabetes mellitus (T2DM). After T2DM induction by high-fat diet and a single streptozotocin injection (25 mg/kg b. w.), male Wistar rats were treated with/without VD (1,000 IU/kg b. w., 30 days). Oxidative stress/inflammation and NOS/NO were assessed by flow cytometry, RT-qPCR, western blotting, and ELISA. A 3.3-fold decrease in serum 25(OH)D_3_ was established in diabetic rats, suggesting their VD deficient status. T2DM was associated with excess reactive oxygen species (ROS; 2.4-fold) and NO (2.5-fold) production in hepatocytes paralleled by elevated levels of myeloperoxidase (1.7-fold), carbonylated (2.8-fold) and nitrotyrosylated (1.7-fold) proteins in liver tissue vs. control, indicative of oxidative-nitrosative stress. Low-grade inflammation in diabetic liver was confirmed by increased NF-κB transcriptional activity (1.24-fold) and mRNA expression of proinflammatory cytokines TNF-α (3.5-fold) and IL-1β (2.2-fold) with alleviating mRNAs of anti-inflammatory cytokines IL-4 (1.7-fold) and IL-10 (2.6-fold), while TGF-β1 expression raised 1.4-fold vs. control. Higher iNOS and eNOS mRNAs (2.7- and 3.3-fold, respectively) and protein (2.1- and 3.2-fold, respectively) levels, as well as NOS activity (1.6-fold) were found in diabetic liver. VD supplementation restored 25(OH)D_3_, partially normalized NF-κB transcriptional activity and pro/anti-inflammatory cytokines, lowered hepatocellular ROS/NO, and oxidative protein modifications. However, VD had no effect on eNOS, IL-10 and TGF-β1 mRNAs. It also led to a further increase in myeloperoxidase, eNOS and iNOS proteins and NOS activity compared to diabetes. In conclusion, abnormal oxidative metabolism in T2DM is associated with enhanced NF-κB/NOS/NO response, which can be partially attenuated by VD treatment via normalization of pro-oxidative/pro-inflammatory processes. The paradoxical sustained increase in NOS expression in the presence of VD antioxidant activity likely improves hepatocellular NO bioavailability, ultimately reducing T2DM-associated liver injury.

## Graphical Abstract

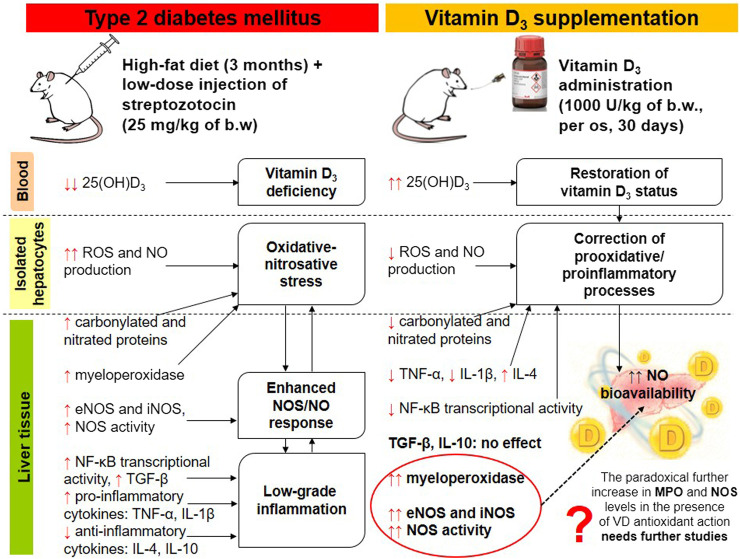

## Impact statement

Progression of liver damage associated with type 2 diabetes mellitus (T2DM) is a major health concern, but the underlying mechanisms remain unclear. Several epidemiological studies have highlighted a significant association between low vitamin D3 levels and an increased risk of metabolic dysfunction-associated steatotic liver disease (MASLD). We hypothesized that excessive NO synthesis could exert protective or detrimental effects on hepatocellular function depending on the intensity of prooxidant and/or proinflammatory processes in the liver in diabetes. Vitamin D3 may increase NO bioavailability by modulating oxidative metabolism and pro-/anti-inflammatory status. Therefore, we investigated for the first time, to our knowledge, the regulatory effects of vitamin D3 on T2DM-induced experimental uncoupling of NO synthase (eNOS, iNOS) expression and NO generation in relation to hyperglycemic state, oxidative stress and chronic inflammation. The obtained findings may have novel implications for preventive strategies against diabetes-induced T2DM and its associated liver complications.

## Introduction

Type 2 diabetes mellitus (T2DM) is currently recognized as a global non-infectious epidemic [1]. The number of adults worldwide suffering from the disease is estimated to reach 650 million by 2045. Hyperglycemia, as a consequence of T2DM, leads to the development of various comorbidities, such as glycemic hepatopathy and metabolic dysfunction-associated steatotic liver disease (MASLD) [2]. According to multiple clinical studies, the prevalence of MASLD is up to 60%–70% of all individuals with type 2 diabetes [3]. It has been shown that the accumulation of triacylglycerols in the liver is an important and early sign of metabolic disorder. At the cellular level, liver damage in T2DM involves glycotoxic and oxidative stress, impaired autophagy and unfolded protein response in the endoplasmic reticulum (ER) during ER stress, alongside with mitochondrial dysfunction [4–6]. In turn, various types of stress can lead to the development of inflammation, induction of apoptotic cell death and enhancement, especially against the background of fibrotic processes, of liver cell proliferation due to activation of the nuclear factor kappa B (NF-κB) signaling pathway [7, 8].

Metastable free radical nitric oxide (NO) production is associated with MASLD and is essential for adequate glucose and lipid homeostasis [9, 10]. This gaseous substance is known to be synthesized by nitric oxide synthases (NOSs). Three isoforms with distinct functions have been identified for the NOS enzyme, namely neuronal (NOS-1; nNOS), endothelial (NOS-3; eNOS), and inducible (NOS-2; iNOS) [11]. Both neuronal and endothelial isoforms generate low levels of nitric oxide as a signaling molecule and are Ca^2+^-dependent. Instead, iNOS activation occurs at the transcriptional level under the influence of endotoxins or inflammatory cytokines, particularly in cells such as macrophages and neutrophils, and is independent of cytoplasmic Ca^2+^ levels. Given that iNOS is a highly yielding enzyme that generates toxic amounts of nitric oxide, it represents a crucial component of the antiparasitic/antimicrobial and antitumor activities of the innate immune system. Nitric oxide is also an important regulator and mediator of numerous processes in the nervous and cardiovascular system. In particular, relaxation of vascular smooth muscles, leading to arterial vasodilation, occurs due to binding to the heme of cytosolic guanylate cyclase. Activation of the latter promotes an increase in the intracellular level of cyclic guanosine-3′,5′-monophosphate (cGMP). In turn, activated cGMP-dependent protein kinase phosphorylates the myosin phosphatase-targeting subunit and the inositol triphosphate receptor-associated cGMP kinase substrate, ultimately resulting in vascular smooth muscle relaxation [12]. Nitric oxide can also act in a cGMP-independent manner, for example, by altering the activity of proteins by covalent nitrosylation of thiol groups of specific cysteine residues in their molecules [13].

In addition to mediating normal functions, NO is involved in a variety of pathophysiological conditions such as hypertension, stroke, neurodegenerative diseases, septic shock, diabetes mellitus and its complications, etc. [9, 14]. Nitric oxide has a significant impact on the liver, playing a pivotal role in regulating metabolism and the immune response [15]. Liver functions depend on complex interactions of heterogeneous subpopulations of its cells. Hepatic nitric oxide is produced primarily by eNOS and iNOS and is responsible for most of the physiological and pathophysiological reactions involving the liver. While nitric oxide formed by eNOS exhibits mainly protective functions in the liver due to the regulation of blood flow and cell interactions, NO synthesized by iNOS can exhibit both protective and damaging effects on liver homeostasis [15]. Experimental models have shown that hepatocytes produce iNOS in response to inflammatory cytokines [16]. Immunohistochemical methods have shown that the eNOS isoform is detected in endothelial cells of arteries and terminal veins of the liver, sinusoids, and in epithelial cells of the bile ducts. Distinct iNOS expression can be observed in the periportal region with decreasing intensity towards the perivenous areas of the hepatic ducts [17]. Liver cells can be exposed to the action of NO generated by neighboring Kupffer cells, endothelial cells, as well as to the influence of autoendogenous NO. It has been shown that iNOS expression is also observed under physiological conditions in the liver and, possibly, through this mechanism, hepatocytes control the degree of apoptosis [18]. Since NO is a multifunctional signaling molecule that plays a striking role in health and disease, adverse changes in the NO system caused by diabetes may be considered as an important link between the diabetic state and liver pathology.

Although there are currently no pharmacological agents specifically approved for the treatment of T2DM-associated liver disease, numerous drug candidates are being studied in clinical trials to address lipotoxic liver injury and insulin resistance, inflammation, fibrosis, and metabolism on a case-by-case basis.

A limited amount of experimental and clinical data is available on the hepatoprotective effect of vitamin D_3_ (cholecalciferol) in T2DM-associated chronic liver diseases [19, 20]. Traditional concepts of the regulatory role of vitamin D_3_ in bone remodeling and mineralization have currently been supplemented by new data that expand the understanding of the pleiotropic effects of this bioactive steroid, which regulates various pathways of cellular metabolism, exhibits anti-inflammatory, immunomodulatory effects and has a powerful impact on cell proliferation, differentiation and death [19–21]. This compound accomplishes its biological effects through the mechanism of hormonal action of the bioactive cholecalciferol derivative, 1α,25-dihydroxyvitamin D_3_ (1α,25(OH)_2_D_3_, calcitriol), which is mediated by a specific vitamin D_3_ receptor (VDR). The latter serves as a transcription factor in regulating the expression of numerous genes [22]. The molecular mechanisms of vitamin D_3_ action in the diabetic liver remain largely unclear. However, it can be speculated that its hepatoprotective efficacy is, at least in part, mediated by the regulation of oxidative-nitrosative stress, NF-κB-associated inflammation, and cell proliferation/death [21, 23]. Vitamin D_3_ was shown to be a promising pharmacological agent for the treatment of metabolic and cardiovascular disturbances, and nitric oxide may play an essential role in mediating these effects [24, 25].

As NO signaling pathway is not only affected by vitamin D_3_ levels but also closely related to MASLD through a series of mechanisms, such as pro/anti-inflammatory imbalance, immune disorders, and oxidative stress, the effect of cholecalciferol on T2DM-elicited liver abnormalities may be achieved via regulation of the NO system. The trial was therefore performed to explore the effects of vitamin D_3_ on hepatocellular oxidative-nitrosative stress and NF-κB-mediated expression of pro/anti-inflammatory cytokines in association with the levels of NO synthases (eNOS, iNOS) and nitric oxide generation in experimental T2DM.

## Materials and methods

### Experimental animals and treatments

We conducted an investigator-blinded, vehicle-controlled study on 35 male Wistar rats weighing 202.5 ± 15.8 g. All rats underwent a 1-week adaptation period to the animal facility environment (temperature +22 ± 2°C, air humidity 50–60%, natural day/night cycle). Food and water were available freely. At the beginning of the experiment, the first random allocation of the animals to two groups was performed: (1) the control rats (n = 10) without somatic pathology, kept on a standard diet for rodents (REZON-1, Ukraine); (2) the T2DM group (n = 25) – animals with experimental T2DM induced by keeping them for 60 days on a homemade high-fat diet (HFD) followed by a single administration of streptozotocin (STZ; Sigma-Aldrich, USA) at a low dose. The HFD was a mixture of a standard vivarium diet (REZON-1, Ukraine) with the addition of vitamins and minerals (based on the daily requirement for animals) – 54%, pork lard prepared by rendering visceral fat – 25%, fructose (Golden Pharm, Ukraine) – 20%, supplemented with bile acids – 1% (Pharma Cherkas, Ukraine). Baseline 25-hydroxyvitamin D_3_ (25(OH)D_3_) levels were measured in all animals in both groups. On day 61 of high-fat diet, rats were intraperitoneally injected with freshly prepared STZ solution after 12 h of fasting (25 mg/kg b.w. dissolved in 0.5 mL of 0.1 M citrate buffer, pH 4.5). Group 1 received an equal (0.5 mL) volume of vehicle (citrate buffer) treatment intraperitoneally. On the 75th day of experiment, the glucose levels were measured by OneTouch Select glucometer (LifeScan Europe GmbH, Switzerland). From the second part of the experiment, we had to exclude 9 animals without or with mild features of diabetes (fasting blood glucose level less than 7.5 mmol/L). At the final stage of the trial, the following groups of rodents were formed: (1) control (n = 7), (2) diabetic (T2DM; n = 7) and (3) diabetic, whose animals were orally (through a gavage) administered 0.1 mL of a solution of vitamin D_3_ (cholecalciferol; SigmaAldrich, USA) in sunflower oil at a dose of 1,000 IU/kg of b. w. daily for 30 days (T2DM + VD; n = 7). The baseline fasting blood glucose levels in the experimental groups were 4.40 ± 0.15, 10.35 ± 1.93 and 10.51 ± 2.02 mmol/L, respectively. Groups 1 and 2 were orally given an equal (0.1 mL) volume of the vehicle (sunflower oil). After the final group formation, all animals were transferred to a standard diet. On day 105, an intraperitoneal insulin tolerance test (ipITT) was performed. The rats were then decapitated under deep ether anesthesia. Blood samples were collected and livers were excised for further analysis. Serum was obtained by centrifugation of whole blood at 3,000 g (+4°C) for 10 min.

All animal procedures were performed in accordance with bioethical principles and international standards concerning animal welfare, including the European Convention for the Protection of Vertebrate Animals Used for Experimental and Other Scientific Purposes (Strasbourg, France; 1986) and the Guidelines for Bioethical Evaluation of Preclinical and Other Scientific Research Conducted on Animals (Kyiv, Ukraine; 2006).

### Assessment of intraperitoneal insulin tolerance test

Experimental rats were fasted for 12 h before performing the intraperitoneal insulin tolerance test. Baseline blood glucose levels (at 0 min) were measured, after that rats were injected intraperitoneally with insulin solution (Actrapid HM Penfill, Novo Nordisk, Denmark) at a dose of 0.7 U/kg b. w. Glucose was monitored at time intervals of 30, 45, 60, 90, and 120 min after insulin administration. Microsoft Excel was used to calculate the total area under the curves in response to insulin injection.

### HOMA-IR determination

HOMA-IR was determined based on the results of fasting blood glucose and insulin measurements using the following formula:

$$\text{HOMA}‐\text{IR}=\text{serum insulin }\left(\text{mmol}/L\right)*(\text{blood glucose }\left(\text{mmol}/L\right)/22.5$$

Serum insulin content was measured by Rat insulin ELISA kit (MBS724709, MyBioSource, USA) following the manufacturer’s protocol.

### Detection of 25-hydroxyvitamin D_3_ content in blood serum

Assessment of vitamin D_3_ status in animals was based on serum 25(OH)D_3_ levels. Vitamin D ELISA kit (DE1971, Demeditec Diagnostics GmbH, Germany) was used to determine 25(OH)D_3_ according to the manufacturer’s instruction.

### Isolation of primary rat hepatocytes and determination of reactive oxygen species and nitric oxide

To isolate primary rat hepatocytes, the oxygenated perfusion solution was slowly infused through the cannulated portal vein and inferior vena cava. After perfusion, the left lobe of the liver was excised and treated with liver digestion medium supplemented with 0.05% collagenase (Sigma, USA) for 1 h (+37°C). Cells were then harvested, washed, and resuspended after centrifugation.

Intracellular generation of reactive oxygen species (ROS) and nitric oxide in isolated hepatocytes was detected using the ROS- and NO-sensitive probes 2′,7′- dichlorodihydrofluorescein diacetate (DCFH-DA; Sigma, USA) and 4,5-diaminofluorescein diacetate (DAF-2DA) (Sigma, USA), respectively. Briefly, hepatocytes (0.5 × 10^6^ cells) were incubated immediately after their isolation in Hank’s balanced salt solution with 10 μM DCFH-DA or DAF-2DA for 30 min (+37°C) in the dark and a humidified atmosphere of 5% CO_2_. After washing by centrifugation and resuspension of the cells, fluorescence was measured using an EPICS XLTM flow cytometer (Beckman Coulter, USA) at 485/530 nm or 495/515 nm (excitation/emission wavelengths for DCFH-DA and DAF-2DA, respectively). Flow cytometric data were then analyzed with FCS Express V3 software. The results were expressed in arbitrary units as the relative fluorescence of the samples compared to the control.

### Nitric oxide synthase activity quantitative assessment

Total nitric oxide synthase activity was determined in liver tissue samples using the NOS Assay Kit (MAK532-1KT, Sigma-Aldrich, USA) in accordance with the manufacturer’s instructions. In brief, liver samples were homogenized in 1x PBS (pH 7.4) and centrifuged at 10,000g (+4°C) to obtain supernatants for NOS activity assay. The step of nitric oxide formation in the NOS reaction of the samples was followed by nitrate reduction to nitrite using the Griess method. The OD was read at 500–570 nm (peak 540 nm).

### Quantitative real-time polymerase chain reaction (RT-qPCR)

Total RNA was isolated from rat liver tissue using the NucleoZOL^®^ single-phase RNA purification kit (Macherey-Nagel, Germany). The extracted RNA was then reverse-transcribed into cDNA with the iScript gDNA Clear cDNA synthesis kit (Bio-Rad, USA). The following primers for RT-qPCR: TGF-β1-Forward: 5′-TGC​TGA​GAA​AAA​GCA​GCA​GA′, TGF-β1Reverse: 5′-AGT​ACG​TCG​TTG​CAG​ATG​TC-3′; TNF-a-Forward: 5′TCA​GCG​AGG​ACA​CCA​AGG-3′, TNF-a-Reverse: 5′-CTC​TGC​CAG​TTC​CAC​ATC​TC-3′; IL-1β-Forward: 5′-AAT​CTC​ACA​GCA​GCA​TCT​CG-3′, IL-1β-Reverse: 5′CAT​CAT​CCC​ACG​AGT​CAC​AG-3′; IL-4-Forward: 5′-GAT​GTA​ACG​ACA​GCC​CTC​TG-3′, IL-4-Reverse: 5′-TGG​TAC​AAA​CAT​CTC​GGT​GC-3′; IL-10-Forward: 5′-ATT​TTA​ATA​AGC​TCC​AAG​ACA​AAG​G-3′, IL-10-Reverse: 5′-GGA​GAG​AGG​TAC​AAA​CGA​GG-3′; NOS2 (iNOS)-Forward: 5′-TTG​GTG​AGG​GGA​CTG​GAC​TTT-3′, NOS2 (iNOS)-Reverse: 5′-TGA​AGA​GAA​ACT​TCC​AGG​GGC-3′; NOS3 (eNOS)-Forward:5′-GTTGACCAAGGCAAACCACC-3′, NOS3 eNOS-Reverse:5′-GCTGACTCCCTCCCAGTCTA-3′; β-actin-Forward: 5′-CCG​TGA​GTT​AGG​TCG​AGC-3′, βactin-Reverse: 5′-GTA​CAT​GCA​GCC​TTC​GTT​G-3′; Mrpl32-Forward: 5′- CAA​AAA​CAG​ACG​CAC​CAT​CG-3′, Mrpl32-Reverse: 5′-GCT​TCA​GGT​GAC​CAC​ATT​CA- 3′. PCR was performed using a SsoAdvanced Universal SYBR Green Supermix kit (Bio-Rad, USA) by CFX Opus 96 Real-Time PCR System (Bio-Rad, USA). Expression levels of β-actin and Mrpl32 were used as double internal standards. Each experiment was performed in triplicate.

### Western blot analysis

The relative levels of target proteins (MPO, iNOS, eNOS, protein carbonyl and 3-nitrotyrosine groups) were determined by western blot analysis. Protein lysates were prepared from liver samples in RIPA lysis buffer containing a cocktail of protease and phosphatase inhibitors (Sigma-Aldrich, USA) according to a standard protocol with subsequent sonication on ice and final addition of Laemmli buffer. Depending on the molecular weight of the target proteins, lysate proteins (60 μg per track) were separated by 10%–15% SDS-PAGE. After transferring the proteins to a nitrocellulose membrane, it was blocked with 5% skim milk or BSA for 1 h and then incubated overnight (+4°C) with the corresponding primary antibodies: MPO (1:1000; ab208670, Abcam, UK); 3-nitrotyrosine (1:500; A21285, Invitrogen, Thermo Fisher Scientific, USA); iNOS (1:500; ab15323, Abcam, UK), eNOS (1:500; ab76198, Abcam, UK). Afterwards, the membranes were incubated for 1 h at room temperature with the appropriate secondary antibodies conjugated with horseradish peroxidase (HRP) and then developed with HRP substrate-specific chemiluminescent agents, namely p-coumaric acid (Sigma-Aldrich, USA) and luminol (AppliChem GmbH, Germany). To measure protein carbonyl groups generated by protein oxidation, a Protein Carbonyl Assay Kit (ab178020, Abcam, UK) for western blot was used according to the manufacturer’s protocol. Quantitation of immunoreactive signals was performed using Gel-Pro Analyzer v3.1 software and corrected for the corresponding β-actin levels, which were determined with anti-β-actin antibody (1:10000; A3854, Sigma-Aldrich, USA).

### NF-κB transcriptional activity

NF-κB p65 transcriptional activity was measured by a sensitive ELISA-based method using the NF-κB p65 Transcription Factor Assay Kit (ab133112, Abcam, UK). Briefly, freshly isolated rat liver nuclear extracts were added to the wells of a 96-well plate, incubated overnight at +4°C and washed. To determine the amount of NF-κB p65 bound to the NF-κB response elements of double-stranded DNA adsorbed at the bottom of the wells, samples were incubated for 1 h with NF-κB p65 primary antibody. After washing and subsequent 1 h incubation with HRP-conjugated secondary antibody followed by the development solution (for 45 min), OD (450 nm) was analyzed using a microplate reader.

### Statistical analysis

We expressed the obtained data as the mean ± standard deviation (SD). For comparison of two experimental groups, Student's t-test was used, and one-way ANOVA followed by Tukey’s post-hoc test was used to compare more than two groups. Differences were considered significant at p < 0.05.

## Results

### Vitamin D_3_ partially improves hyperglycemia and insulin resistance in type 2 diabetes

**FIGURE 1:**
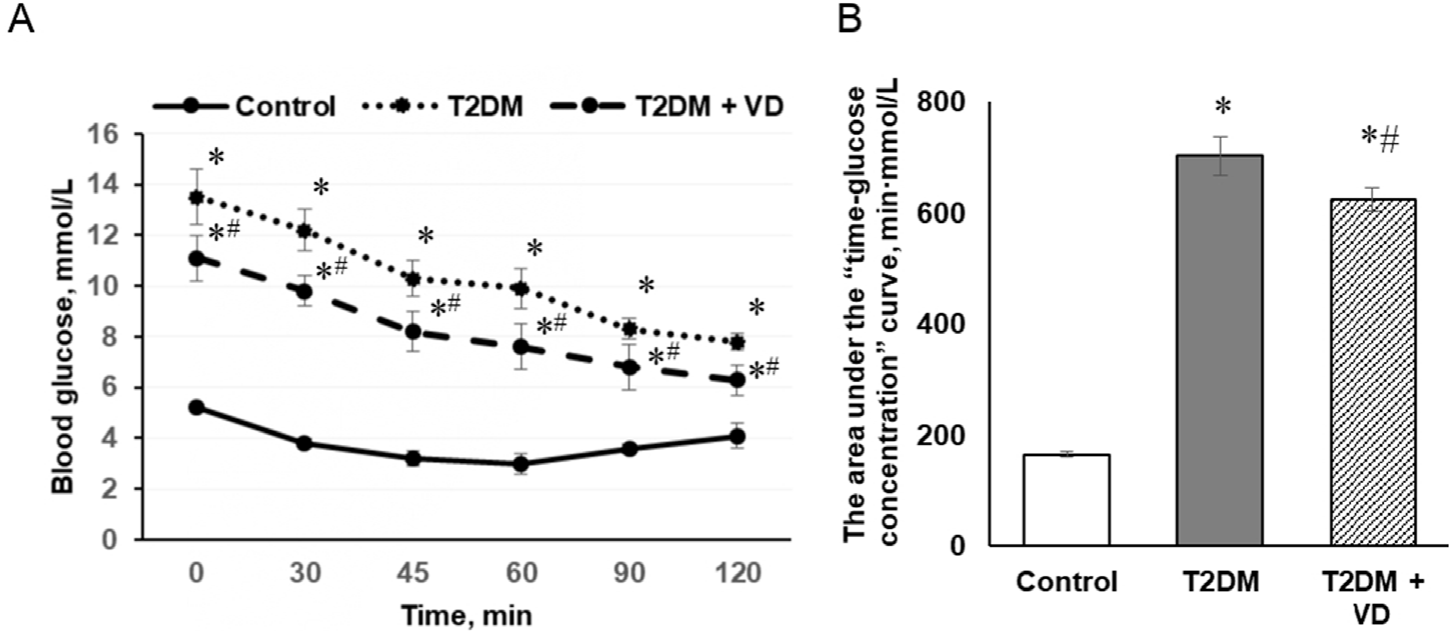
The effect of vitamin D_3_ treatment (1,000 IU/kg body weight, 30 days) on the insulin tolerance test in male Wistar rats after 3-month HFD and a single injection of streptozotocin (25 mg/kg). **(A)** The concentration of blood glucose in dynamics after intraperitoneal injection of insulin (0.7 units/kg) to rats; **(B)** Bar graphs represent the area under the “time-glucose concentration” curve. The data are presented as means ± SD, n = 7; *p < 0.05, the T2DM group vs. the control group, ^#^ *p* < 0.05, the T2DM + vitamin D_3_ group vs. the T2DM group.

In experimental type 2 diabetes, we observed a significant deterioration of carbohydrate homeostasis, which was confirmed by more than twofold increase in blood glucose levels (Table 1) and the development of insulin resistance (Figures 1A,B). Insulin resistance was assessed using an intraperitoneal exogenous insulin tolerance test. In diabetic animals, basal blood glucose levels after an overnight fast were shown to increase markedly at all time points following insulin administration (0.7 U/kg). Blood glucose in control rats after insulin injection reached a minimum at 45 min (2.63 mmol/L), while in animals with T2DM it was minimal only after 120 min (7.50 mmol/L). Calculations based on the ipITT revealed a nearly fourfold increase in the area under the curve in diabetes compared to the control group (Figure 1B), indicating a significant slowdown in glucose uptake by peripheral tissues as a result of insulin resistance. Furthermore, we found a 4.2-fold enhancement of HOMA-IR in rats fed a high-fat diet and given a single injection of STZ compared to control animals (Table 1). This corresponds to increased resistance of cells and tissues to insulin and the progression of T2DM. Pharmacotherapy with vitamin D_3_ (1000 IU/kg b.w., for 30 days), partially recovering the sensitivity of peripheral tissues to insulin, contributed to enhanced tissue utilization of glucose, which was manifested by mild compensation of hyperglycemia in animals with T2DM. Thus, the basal glucose level decreased by 16% in vitamin D_3_-treated rats (Table 1). The study of glycemic response during the ipITT showed a 12% decrease in the area under the curve after cholecalciferol administration compared to the diabetic group of animals (Figure 1B). Vitamin D_3_ also partially diminished the HOMA-IR index (by 33%).

**TABLE 1:** Body weight, fasting blood glucose and HOMA-IR in rats with T2DM and after vitamin D_3_ administration (1,000 IU/kg body weight, 30 days).

Parameter	Control	T2DM	T2DM + Vitamin D3
Weight, g	369.7 ± 12.7	383.4 ± 12.2	372.3 ± 10.5
Blood glucose, mmol/L	5.32 ± 0.12	13.10 ± 1.44*	11.00 ± 0.61^#^
HOMA-IR	0.70 ± 0.05	2.93 ± 0.17*	1.95 ± 0.12^#^

*Note*. All data are presented as means ± SD, n = 7; *p < 0.05, the T2DM group vs. the control group, ^#^ *p* < 0.05, the T2DM + VD group vs. the T2DM group.

### Experimental type 2 diabetes is accompanied by vitamin D_3_ deficiency

**FIGURE 2:**
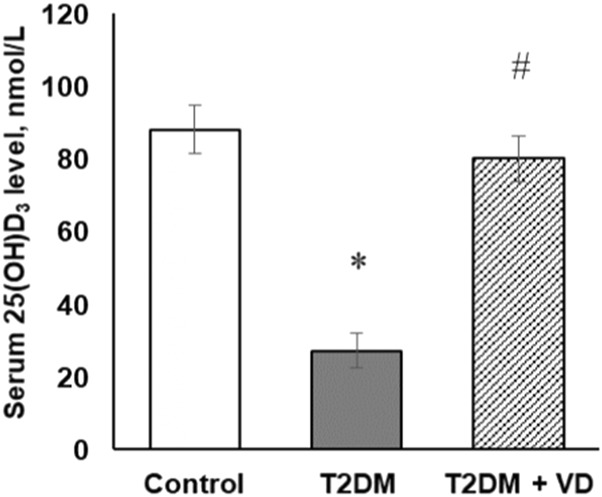
The serum level of 25-hydroxyvitamin D_3_ (25(OH)D_3_) of diabetic rats and after supplementation of vitamin D_3_ (1,000 IU/kg body weight, 30 days). The data are presented as means ± SD, n = 7; *p < 0.05, the T2DM group vs. the control group, ^#^ *p* < 0.05, the T2DM + vitamin D_3_ vs. the T2DM group.

Maintaining adequate vitamin D_3_ status prevents the development of numerous diseases, including both types of diabetes [26]. Therefore, we assessed serum 25(OH)D_3_ levels as a main marker of vitamin D_3_ bioavailability in animals. Measurement of baseline 25(OH)D_3_ during random assignment of animals to control and diabetic groups before the onset of diabetes revealed no between-group differences. All animals were characterized by vitamin D_3_ sufficiency (92.8 ± 6.3 nmol/L). Such homogeneity of the initial values of 25(OH)D_3_ indicates the absence of systemic disturbances in the metabolism of vitamin D_3_ and its bioavailability before experimental interventions, which allowed us to reliably assess the contribution of each factor (vitamin D_3_ deficiency, prooxidation/inflammation, vitamin D_3_ supplementation) to T2DM-induced changes in the liver. It was found that T2DM progression is accompanied by a threefold decrease in circulating 25(OH)D_3_ compared to control rats, indicating the development of a D_3_-deficient state in diabetic animals (Figure 2). Therapeutic intervention with vitamin D_3_ in animals with type 2 diabetes almost completely normalized serum 25(OH)D_3_ levels.

### Vitamin D_3_ inhibits reactive oxygen species formation and oxidative protein modifications in type 2 diabetic liver

**FIGURE 3:**
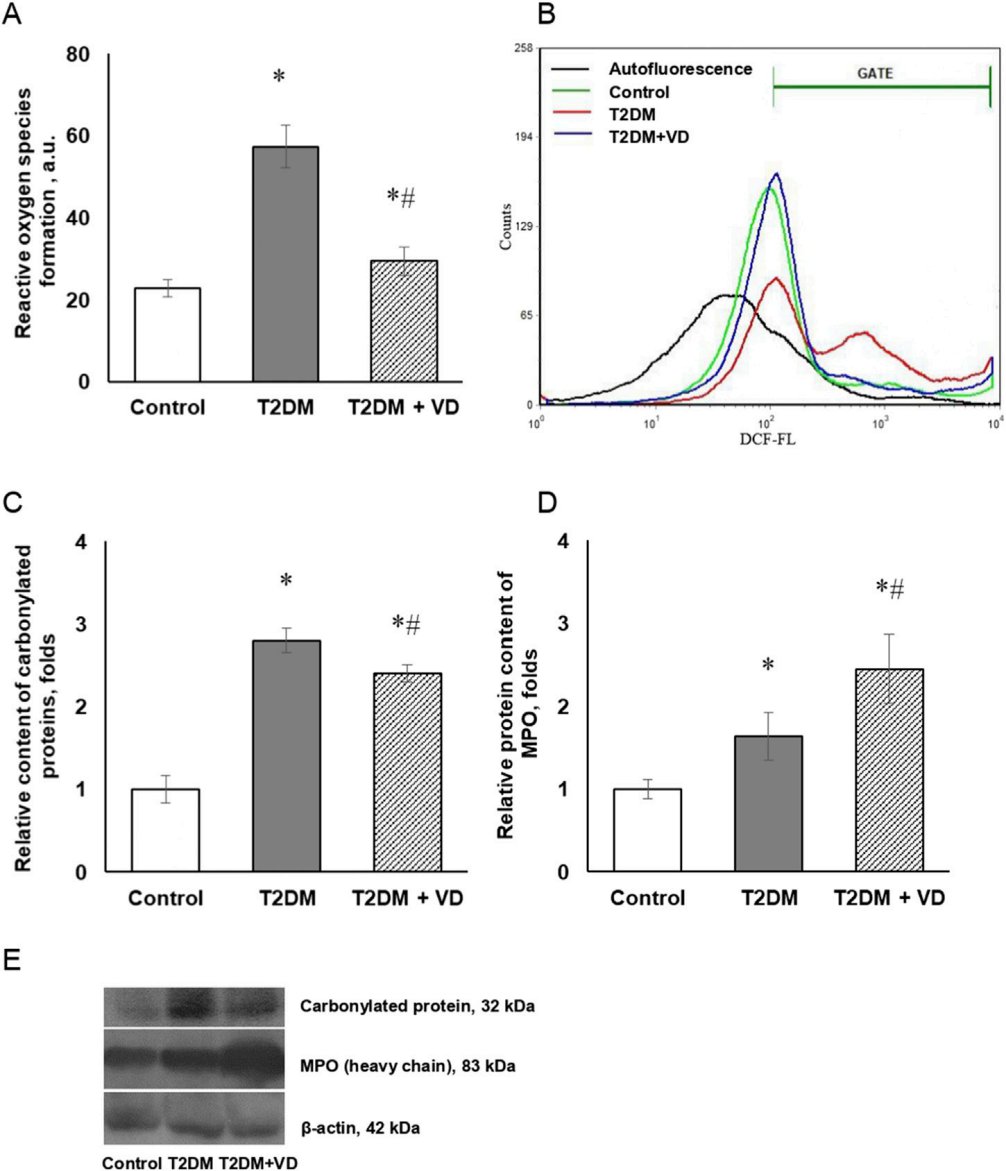
Reactive oxygen species formation and oxidative protein modifications in type 2 diabetic liver. Quantification of DCFH-DA oxidation in isolated rat hepatocytes documented by flow cytometry analysis **(A)** and representative histograms of DCF fluorescence **(B)** are shown. Relative content of carbonylated proteins **(C)** and MPO **(D)** in the liver of diabetic rats and after supplementation of vitamin D_3_ (1,000 IU/kg body weight, 30 days) was determined by western blot analysis. Representative immunoblots **(E)** are shown. The data are presented as means ± SD, n = 7; *p < 0.05, the T2DM group vs. the control group, ^#^ *p* < 0.05, the T2DM + vitamin D_3_ vs. the T2DM group.

Excessive formation of ROS and reactive nitrogen species (RNS) and the associated oxidative-nitrosative stress are currently considered as universal mechanism of detrimental changes in diabetes-related complications [27]. In order to clarify the possible contribution of prooxidative processes to hepatocellular dysfunction induced by type 2 diabetes, the level of free radical generation in primary hepatocyte culture, using the ROS-sensitive probe DCFH-DA, was investigated. We found that T2DM leads to elevated ROS formation in isolated hepatocytes (Figures 3A,B). This is evidenced by an increase in the fluorescence intensity of the oxidized form of the probe, DCF, by more than twofold compared to control animals. Pharmacotherapeutic application of vitamin D_3_ reduced the level of hepatocellular ROS generation almost to the control level, which is consistent with available data on the protective antioxidant effect of this compound [28].

It is known that T2DM-associated hyperglycemia significantly increases the rate of formation of oxidatively modified (carbonylated) proteins, which reliably reflects the degree of oxidative stress. Intensification of carbonylated protein formation may be an important indicator of damage to cell structures in liver pathology. In this regard, we studied the level of covalently modified (carbonylated) proteins in the liver of diabetic animals that was 2.8-fold elevated compare to intact controls (Figures 3C,E). Cholecalciferol supplementation caused a moderate decrease in the level of protein carbonylation compared to diabetic rats.

An important enzyme that may play a role in oxidative stress is myeloperoxidase (MPO). Being directly involved in the antimicrobial activity of neutrophils and the defense of the body against various pathogens, primarily through participation in phagocytosis, MPO is released from leukocytes and catalyzes the formation of several reactive compounds, including hypochlorous acid (HOCl) and hypothiocyanic acid [29]. These compounds are involved in oxidative stress and post-translational modification of target proteins. Despite the importance of the antimicrobial function of innate immunity, unregulated release of MPO leads to tissue damage. Determination of the amount of MPO protein, one of the common markers of oxidative stress/inflammation, in the diabetic liver showed its increase by 1.7 times compared to the control (Figures 3D,E). An unexpected observation was that the administration of cholecalciferol further increased the expression of this prooxidant enzyme. The inconsistency of the obtained results necessitates the need for further studies to clarify the full potential of vitamin D_3_ in regulating myeloperoxidase activity in the studied pathology.

### Vitamin D_3_ attenuates nitric oxide and 3-nitrotyrosine levels while increasing iNOS and eNOS protein synthesis and NOS activity in diabetic rat liver

**FIGURE 4:**
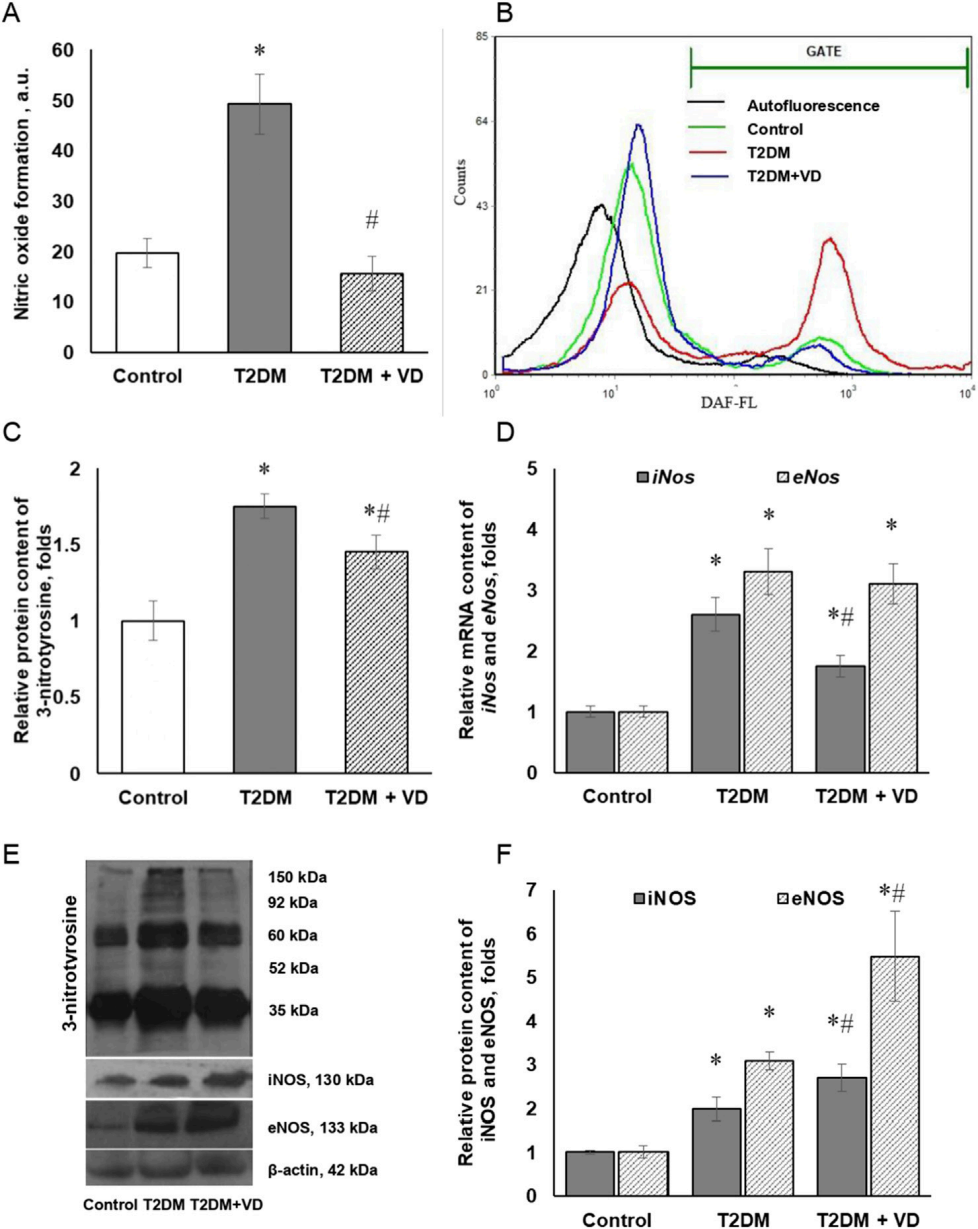
Nitric oxide formation in isolated rat hepatocytes in type 2 diabetic liver and effect of vitamin D_3_ administration. Quantification of DAF oxidation documented by flow cytometry analysis **(A)** and representative histograms of DAF fluorescence **(B)** are shown. Relative protein level of 3-nitrotyrosine **(C)**, iNOS and eNOS mRNA **(D)** and protein content **(F)**, as well as their representative immunoblots **(E)** are shown in the liver of diabetic rats and after vitamin D_3_ treatment (1,000 IU/kg body weight, 30 days). The data are presented as means ± SD, n = 7; *p < 0.05, the T2DM group vs. the control group, #p < 0.05, the T2DM + vitamin D_3_ group vs. the T2DM group.

Using the NO-sensitive probe DAF-2DA, we demonstrated that T2DM induces a more than twofold elevation of NO synthesis in isolated hepatocytes (Figures 4A,B). Vitamin D_3_ partially suppressed the intensity of nitric oxide formation. In accordance with the synergistic ability of NO and ROS to covalently modify protein molecules, tyrosine residues in the latter are sensitive targets for the active nitrating agent, peroxynitrite. Thus, the content of 3-nitrotyrosine is considered as a specific marker of peroxynitrite activity and associated oxidative-nitrosative stress [30]. Figures 4C,E shows immunoblotograms of the relative content of nitrated proteins in the liver tissue of diabetic animals, as well as under the action of vitamin D_3_. In the liver of diabetic rats, an increased content of protein macromolecules with 3-nitrotyrosine moieties (1.7 times) was found compared to the control group, clearly highlighting the development of oxidative-nitrosative stress. Cholecalciferol supplementation reduced the content of nitrotyrosylated proteins by 20% compared to the diabetic group. Remarkably, the findings of other authors showed a link between vitamin D_3_ deficiency in rats and the development of nitrosative stress, associated with increased formation of 3-nitrotyrosine [31].

**FIGURE 5:**
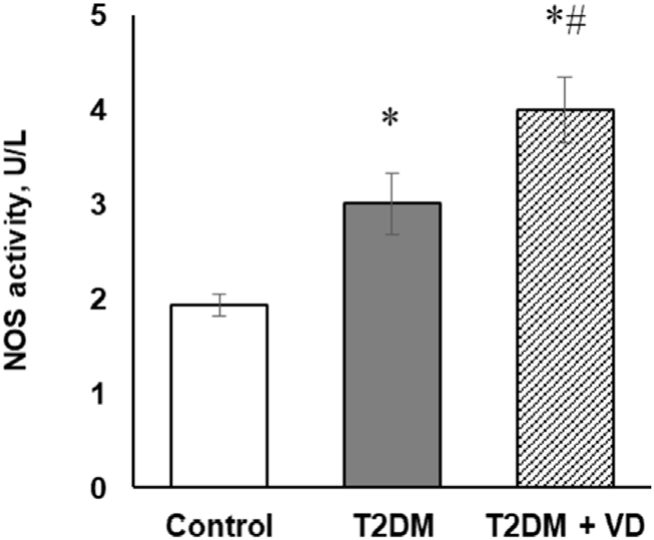
The enzymatic NOS activity in the liver of diabetic rats and after supplementation of vitamin D_3_ (1,000 IU/kg body weight, 30 days). The data are presented as means ± SD, n = 7; *p < 0.05, the T2DM group vs. the control group, #p < 0.05, the T2DM + vitamin D_3_ group vs. the T2DM group.

In the liver, the inducible and endothelial NO synthase isoforms are the key enzymes responsible for nitric oxide synthesis. Diabetes-associated liver disorders were characterized by a strong elevation of their expression at both transcriptional and translational levels. We showed, respectively, a 2.7- and 3.3-fold upregulation of iNOS and eNOS mRNA expression (Figure 4D) in the liver of diabetic rats that paralleled by an equally profound increase in their protein levels (2.1- and 3.2-fold, respectively), Figures 4E,F. The upregulated NOS levels align with our findings of increased NO production as well as the formation of 3-nitrotyrosine residues within oxidatively modified proteins. These changes were also found to positively correlate with the enhancement of overall NOS enzymatic activity (1.5-fold) in the liver tissue of diabetic rats (Figure 5). While partially abrogating T2DM-associated increase in iNOS mRNA (1.58-fold) and leaving eNOS mRNA expression unchanged (Figure 4D), vitamin D_3_ supplementation contributed to a further significant enhancement of iNOS and eNOS protein synthesis (1.45- and 1.92-fold, respectively) in comparison with the diabetic state (Figures 4E,F). As shown in Figure 5, the discovered increase in iNOS and eNOS protein synthesis correlated with the elevated NOS enzymatic activity in diabetes and under the influence of vitamin D_3_.

### Vitamin D_3_ hepatoprotective effects involve modulation of pro-inflammatory/ anti-inflammatory cytokine and transforming growth factor expression

**FIGURE 6:**
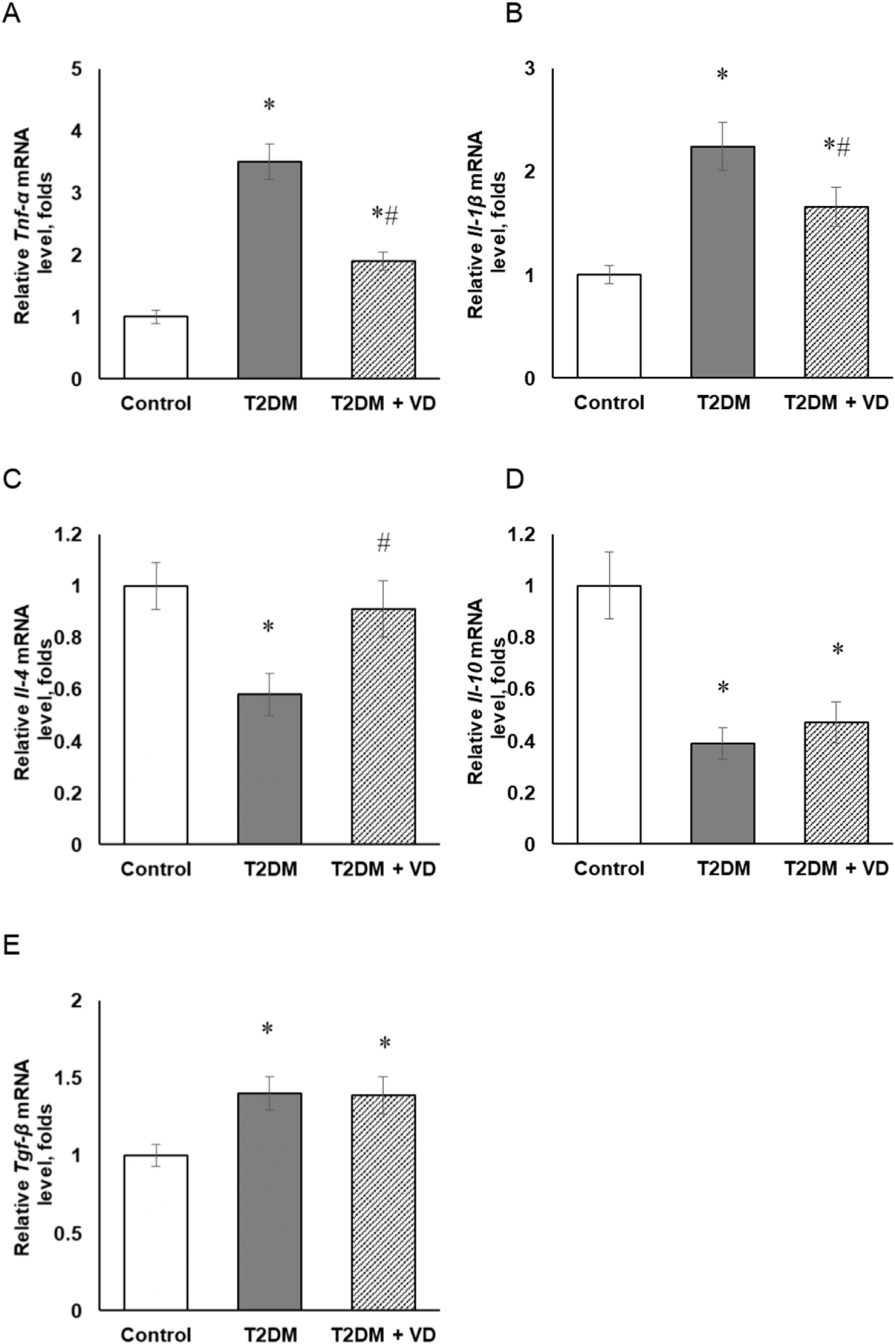
The relative gene expression of pro/anti-inflammatory cytokines in the liver tissue of rats with T2DM and after vitamin D_3_ treatment (1,000 IU/kg body weight, 30 days). Relative mRNA content of *Tnf-α* **(A)**, *Il-1β* **(B)**, *Il-4* **(C)**, *Il-10* **(D)**, and *Tgf-β* **(E)** was determined by RT-qPCR. mRNA levels were adjusted to *β-actin* and *Mrpl3*2 expression. The data are presented as means ± SD, n = 7; *p < 0.05, the T2DM group vs. the control group, ^#^ *p* < 0.05, the T2DM + vitamin D_3_ group vs. the T2DM group.

Сytokines and growth factors are capable of inducing different cellular mechanisms leading to NOS induction. In particular, inflammatory cytokines can act as triggers that switch NO synthesis from constitutive to inducible [15]. Cytokine involvement and disruption of inflammatory cell interactions are also among the “kindling mechanisms” of the concept of eNOS uncoupling in the development of inflammation and vascular dysfunction [12]. We have shown that diabetes-associated upregulation of the iNOS and eNOS mRNA and protein expression (Figure 4), described in the previous section, correlated with significantly higher mRNA transcript levels of pro-inflammatory cytokines such as TNF-α (tumor necrosis factor alpha) and IL-1β (interleukin 1 beta) by 3.5- and 2.2-fold, respectively (Figures 6A,B). Another important pro-inflammatory factor, which belongs to the group of CC-chemokines (β-chemokines), is MCP-1 (monocyte chemoattractant protein), or CCL2 (C-C motif ligand). This bioregulatory compound is the most potent factor of monocyte chemotaxis in mammals that controls the egress of cells from hematopoietic organs and their trafficking to the foci of inflammation [32]. MCP-1 was established to be involved in some mechanisms of insulin resistance, as well as liver fibrosis and a number of other pathologies associated with chronic inflammation. The results of our study indicate a profound increase in the protein content of this pro-inflammatory chemokine in the liver of diabetic rats, while it was not detected at all in control rats under these experimental conditions (data not shown).

The advanced diabetes-evoked synthesis of pro-inflammatory factors could be rescued by the formation of anti-inflammatory cytokines. In light of this, gene expression of such anti-inflammatory cytokines as IL-4 and IL-10, as well as TGF-β1 (transforming growth factor beta) in liver tissue was investigated. Our findings indicate that the diabetic state, against the background of intensified pro-inflammatory cytokines formation, is characterized by a significant 1.72- and 2.56-fold lowering of IL-4 (Figure 6C) and IL-10 (Figure 6D) mRNAs, respectively, with a 1.40-fold increase in the level of TGF-β1 mRNA (Figure 6E). Vitamin D_3_ shifted the pro-inflammatory/anti-inflammatory balance towards the anti-inflammatory profile. The increase in the mRNA expression of IL-10 following vitamin D_3_ administration was accompanied by a decrease in the levels of TNF-α and IL-1β mRNAs and MCP-1 protein. The expression level of IL-4 and TGF-β did not change under the influence of cholecalciferol compared to the diabetic group of animals.

### Nuclear factor κB activation is implicated in type 2 diabetes-induced liver disorder and in the mechanism of hepatoprotection associated with vitamin D_3_ treatment

**FIGURE 7:**
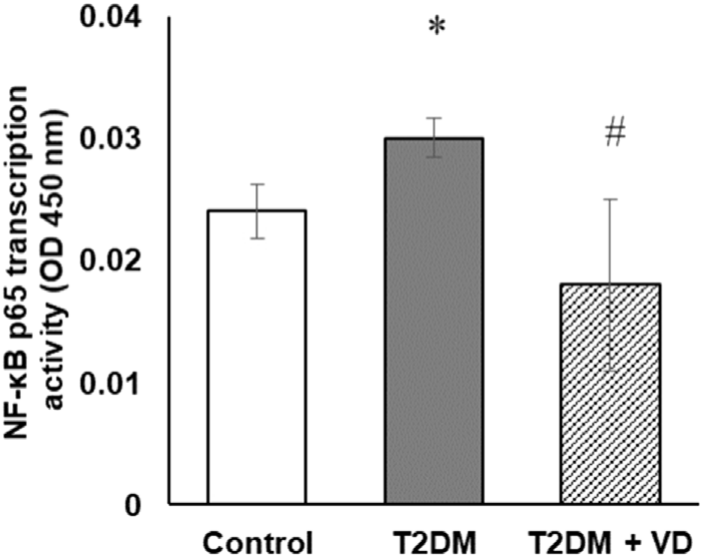
The NF-κB p65 transcription activity in the nuclear fraction of liver extracts of rats with T2DM and after vitamin D_3_ treatment (1,000 IU/kg body weight, 30 days). Data are shown as means ± SD, n = 7; *p < 0.05, the T2DM group vs. the control group, ^#^ *p* < 0.05, the T2DM + vitamin D_3_ group vs. the T2DM group.

Free radicals are known to activate the transcription factor NF-κB regardless of the mechanism and source of their formation [33]. Nuclear factor κB affects the function of many genes responsible for the immune response, inflammation, survival and programmed cell death, as well as for their proliferative activity. In particular, NF-κB has also been shown to indirectly or directly target induction of NOS promoters and downstream expression of NO synthases [34]. To confirm the potential involvement of NF-κB in the mechanism of liver dysfunction associated with T2DM, it was important to investigate the transcriptional activity of NF-κB and its correlation with the delineated changes in the expression of NO synthases and key cytokines involved in inflammation. The transcriptional activity of NF-κB was determined in nuclear extracts of liver cells by the ELISA method according to the level of its binding to the NF-κB-response element of double-stranded DNA fragments. Our study revealed a 24% increase in NF-κB transcriptional activity in diabetes compared to controls (Figure 7). Vitamin D_3_ effectively reduced this activity to near control values.

## Discussion

Oxidative stress, which is associated with a shift in the balance between reactive oxygen/nitrogen species generation and the efficiency of the antioxidant defense system towards the predominance of prooxidant processes, attracts the attention of researchers as one of the cornerstones of undesirable changes in the development of numerous chronic complications of T2DM, and MASLD is no exception. In the present study, we attempted to confirm the hypotheses that unbalanced nitric oxide metabolism in type 2 diabetes mellitus may facilitate and exacerbate oxidative damage to hepatocellular structures, and vitamin D_3_ could be proposed as a therapeutic agent to manage diabetes-evoked liver disorders.

The ability of the NO molecule to exhibit cytoprotective or cytotoxic effects is known to depend on the cell type, its development phase and biochemical potential, as well as on the local concentration of NO and the presence of ROS. As recent studies highlight, the balance between the protective and cytotoxic effects of NO may be determined, among other factors, by the redox status of the cell [35, 36]. The cytotoxicity of the NO seems to be associated with excessive accumulation of superoxide anions in the lesion site, which, interacting with the NO molecule, form a potent mediator of oxidative cellular damage – peroxynitrite [30]. The latter is capable of oxidizing NH- and SH- groups of proteins, lipoproteins, DNA, leading to the loss of the native properties of these biomolecules. Reacting with metal ions, as part of SOD (superoxide dismutase), peroxynitrite causes the production of a reactive and highly toxic nitrosonium ion (NO_2_^+^), which, in turn, forms nitrophenols [37–39]. As a result, the functions of highly specialized protein molecules are disrupted. In the presence of peroxynitrite or its decay product, glutathiyl radical (GS·) is formed and, as a consequence, the latter is converted from an antioxidant into a prooxidant, initiating lipid peroxidation [40]. All this determines the cytotoxic effect of peroxynitrite, leading to cell death through apoptosis or necrosis [30, 38].

The level of 3-nitrotyrosine moieties within protein macromolecules is considered a specific and reliable marker of peroxynitrite activity [41]. The significant accumulation of nitrated proteins in liver tissue that we discovered, associated with overproduction of ROS and NO, along with an increase in the content of proteins with carbonylated groups and myeloperoxidase level, is quite convincing evidence of the development of oxidative-nitrosative stress in T2DM. In addition, increased peroxynitrite formation caused by diabetes most likely reduces the bioavailability of nitrogen oxide and interferes with the physiological effects of the latter in liver cells.

Nuclear factor κB, as a potent cellular redox sensor, appears to be highly sensitive to free radical formation. ROS and NO, regardless of the mechanism and source of their generation, activate NF-κB [33]. It is known that iNOS expression, which is mainly regulated at the transcriptional level, can be directly induced by activated NF-κB through its binding to the iNOS promoter containing NF-κB-binding sequence [42]. Moreover, multiple NF-κB enhancer elements have also been found upstream to the iNOS promoter, conferring inducibility to several signaling molecules [43]. Among such iNOS inducers, NF-κB mediates the expression of a number of growth factors, chemokines, and cytokines [34, 44]. Specifically, iNOS can be activated either by IFN-γ (interferon gamma) signal alone or by TNF-α in the presence of other signals [45, 46]. At the same time, the action of TGF-β is associated with a strong inhibitory effect on iNOS, whereas IL-4 and IL-10 are weak inhibitory signals [47, 48].

In contrast to iNOS, whose production is largely affected at the transcriptional level, constitutively expressed eNOS is subject to subtle post-transcriptional regulation according to its physiological role in signal transduction. These eNOS-mediated mechanisms link NO signaling cascades to its physiological functions in vascular endothelium, including vasorelaxation, angiogenesis, pro/anti-inflammatory balance of endothelial cells, etc. [49]. In particular, eNOS expression is increased by TGF-β, estrogens, and, in some experiments, by exposure of endothelial cells to high glucose concentrations [50, 51]. Several studies have provided evidence that TNF-α and NF-κB activation can repress eNOS synthesis through post-transcriptional destruction of its mRNA without affecting eNOS promoter activity. Mechanistically, this involves NF-κB-dependent formation of specific microRNA targeting eNOS mRNA [52]. Whether activation of the NF-κB is a necessary step for eNOS induction at the transcriptional level is still debatable. However, several experimental studies have demonstrated that NF-κB activation is required for eNOS gene induction, and corresponding binding sites in the iNOS promoter have been identified [53].

Our study further develops the hypothesis that activation of NF-κB-associated signaling pathways may be one of the key events resulting in liver dysfunction at the cellular level in T2DM. In particular, insufficient nitric oxide bioavailability could be compensated by even greater intensification of its production, involving NF-κB-mediated expression of NO synthases and contributing to the formation of a vicious circle of amplified oxidative damage to biomolecules and inflammation with much more dangerous consequences for cellular function. In the present study, we found enhanced NF-κB activity accompanied by upregulated mRNA synthesis of pro-inflammatory cytokines and downregulated mRNA levels of anti-inflammatory cytokines in T2DM. These changes correlated with elevated expression of iNOS and eNOS at both transcriptional and translational levels. The findings are consistent with the data of other researchers on diabetes-evoked expression of the inducible form of NOS [54]. Regarding the expression of eNOS, the available literature data are contradictory and range from upregulated expression of this enzyme to reduced levels [55, 56]. Our results are not in line with the aforementioned role of TNF-induced NF-κB activation, which post-transcriptionally reduces eNOS levels [52]. Rather, they are consistent with the possibility of eNOS promoter activation by NF-κB at the transcriptional level of regulation. Although TGF-β expression increased under our experimental conditions, the level of its production is probably insufficient to effectively counteract the increased synthesis of NOSs.

Notably, nitric oxide is able to inhibit the expression of the iNOS gene by reducing the level of NF-κB in a culture of primary human and rat hepatocytes [57, 58]. These cells can produce NO in large quantities, and such physiological response seems to involve a negative feedback mechanism that serves to prevent unrestrained tissue damage. A similar mechanism is likely also relevant to the regulation of NF-κB activity in vascular endothelium mediated by eNOS. The resulting NO reduces the expression of the NOS genes, limiting the excessive formation of this metabolite under pathophysiological conditions. Apparently, this mechanism of switching off the stimulating effect of NF-κB on gene expression can be impaired under conditions of T2DM-associated liver pathology. Furthermore, it has been shown that NF-κB transcriptional activity largely depends on the intracellular redox state. In particular, the redox state of intracellular glutathione is believed to determine hepatocellular iNOS synthesis both *in vivo* and *in vitro* and this correlates with NF-κB activation [59]. Glutathione depletion prevented iNOS induction in hepatocytes, although this phenomenon has not been established in cells involved in the immune response during inflammation.

Our investigation revealed a significant, but not entirely unambiguous, modulating effect of vitamin D_3_ on the functioning of the NO system in the diabetic liver. Vitamin D_3_ appears to be effective in reducing ROS and NO generation in primary hepatocyte culture, which occurs in parallel with attenuation of 3-nitrotyrosine in liver tissue of diabetic rats.

However, the impact of cholecalciferol on the synthesis of eNOS and iNOS at the translational level, as well as on total NOS activity in liver tissue, was potentiating in relation to the effects of T2DM on these parameters. Although the increased production of nitric oxide by hepatocytes in diabetes was reduced by the action of vitamin D_3_, its enhanced formation is possible due to the contribution of other types of liver cells. However, it remains unclear by which cells this generation of nitric oxide could occur and which NOS isoforms are involved in the intensification of its synthesis. Elevated NO levels are most likely not toxic for cells, because their formation takes place against the background of mitigated oxidative stress under the action of vitamin D_3_. This is evidenced by a decrease in the intensity of ROS/NO generation in hepatocytes, as well as the accumulation of carbonylated and nitrated proteins in diabetic liver tissue after cholecalciferol treatment. Simultaneously, available literature highlights the ability of vitamin D_3_ to positively affect the antioxidant capacity and the redox state of cells, in particular, the glutathione redox system [60]. The delineated upregulation of key NO-generating isoforms of liver NO synthase and an increase in the total activity of NOS are probably aimed at compensating for the reduced bioavailability of nitrogen oxide in diabetes and more effectively accomplishing its physiological functions in hepatocellular signaling.

Indeed, growing line of evidence suggests that NO is an important physiological regulator of hepatocyte function [15–17]. As is known, when hepatocytes are stimulated by inflammatory mediators or glucocorticoids, these cells secrete proteins characteristic of the acute phase of inflammation. It has been shown that NO generated by hepatocytes and resident macrophages (Kupffer cells) reduces the total production of these proteins [61]. Other studies confirm that in hepatocytes nitric oxide exerts an inhibitory effect on glucose metabolism that may have relevance in the context of its antidiabetic action. In particular, *in vitro* experiments have shown significant NO-dependent inhibition of glyceraldehyde-3-phosphate dehydrogenase in the liver of rats expressing high levels of iNOS [62]. The ability of NO to inhibit hepatic gluconeogenesis in cell culture was also demonstrated [63]. Furthermore, hepatocyte-specific eNOS knockout was found to impair the energy-sensing ability of these cells and suppress the activation of the autophagy initiating factor ULK1, indicative of the novel role of eNOS in liver cell mitochondrial adaptation [64]. Higher amounts of NO produced by iNOS or exogenous nitric oxide are known to prevent apoptosis. Stimulated glutathione oxidation is thought to be a possible mechanism by which NO induces Hsp70 protein and provides protection against apoptosis triggered by TNF-α in cultured rat hepatocytes [65]. The ability of nitric oxide to nitrosylate thiols in the active sites of caspases and inhibit their activity is another potential mechanism for the modulation of apoptosis [66]. Finally, some evidence suggests the existence of a negative feedback mechanism by which DNA damage elicited by NO activates p53 expression that, in turn, blocks the iNOS gene promoter and iNOS expression [67].

The positive hepatoprotective effect of vitamin D_3_ in the liver is in line with its inhibitory action on NF-κB transcriptional activity and the activity-dependent expression of pro-inflammatory cytokines. This function, on the one hand, can be mediated by the direct inhibitory effect of cholecalciferol on NF-κB, and also indirectly due to the normalization of oxidative metabolism and the suppression of ROS/RNS formation as strong inducers of this transcription factor. In turn, reduced expression and transcriptional activity of NF-κB attenuates the synthesis of pro-inflammatory cytokines and slows down cytokine-evoked nitric oxide formation. Studies by other authors also show that vitamin D_3_ is a negative regulator of NF-κB transcriptional activity at the genomic level, the effects of which are mediated by vitamin D_3_ receptor [68]. They demonstrated that VDR, through direct interaction with the IKKβ (inhibitory kappa B kinase beta), suppresses the canonical pathway of NF-κB activation. The uncovered discrepancy between iNOS protein and mRNA levels after taking vitamin D_3_, respectively upregulate and downregulated, can be explained by probable changes in the stability of iNOS mRNA and protein at the post-transcriptional and post-translational levels of regulation [12].

We finally turn to the unexpected observation of a sustainable increase in myeloperoxidase protein expression after vitamin D_3_ supplementation, which deserves a more detailed insight. Although the available scientific data generally describes an inverse correlation between 25(OH)D_3_ and myeloperoxidase levels, the detected elevation of MPO expression after vitamin D_3_ treatment was not accompanied by an intensification of oxidative stress/inflammation, so we can conclude that MPO-mediated oxidation may play a minor role in the overall oxidative stress and low-grade metabolic inflammation in the liver of diabetic rats. Presumably, increased MPO synthesis under the influence of cholecalciferol can be associated with the functional activation of non-resident and resident macrophages, whose elevated phagocytic activity can help regenerative processes in liver tissue and improve insulin sensitivity. In particular, several lines of evidence implicate MPO and RNS in the oxidative modification of low-density lipoproteins, leading to their increased uptake by macrophages [69]. This suggestion aligns with our previous findings that vitamin D_3_ may prevent the immunosuppressive effects of glucocorticoids by enhancing the efficiency of oxygen-dependent phagocytic mechanisms in peripheral blood neutrophils and monocytes, thereby increasing the functional activity of phagocytic cells [70]. Thus, the reduction in the propagation of prooxidative/inflammatory processes, in our opinion, may partly justify the stimulation of MPO sufficient to maintain adequate functioning of the innate immune system without MPO-associated tissue damage.

Notably, all metabolic disorders caused by T2DM were observed against the background of vitamin D_3_ deficiency in diabetic rats, which can independently, as an additional pathogenic factor, contribute to the development of liver failure associated with diabetes. It should also be emphasized that, according to the most recent data, a link has been established between vitamin D_3_ deficiency in animals and the propagation of nitrosative stress associated with increased formation of 3-nitrotyrosine in various tissues [31].

We conclude that experimental T2DM is associated with increased ROS and NO formation in primary hepatocyte culture, which is closely related to an increased NF-κB-dependent expression of inflammation markers and NOSs and is accompanied by the intensification of oxidative-nitrosative stress in rat liver. Our findings shed light on the hepatoprotective role of vitamin D_3_ intervention, which modulates NF-kappaB activity and NOS expression/NO production, key hepatocellular responses to inflammatory mediators and oxidative stress associated with T2DM. These changes are likely to improve the bioavailability of NO for liver cells during vitamin D_3_ supplementation. Further studies are required to definitively decipher the true significance of the aggravated stimulation of MPO, iNOS and eNOS synthesis following vitamin D_3_ treatment that could help to better understand its protective effects in diabetes.

## Data Availability

The raw data supporting the conclusions of this article will be made available by the authors, without undue reservation.
